# Conditional cash transfers and gender constructions: A unique dataset on women in Buenaventura, Colombia

**DOI:** 10.1016/j.dib.2020.106290

**Published:** 2020-09-07

**Authors:** Blanca Zuluaga, Daniella Castellanos, Carolina Borda

**Affiliations:** a Economics Department, Icesi University, Colombia; b Social Studies Department, Icesi University, Colombia; c Fundación WWB, Colombia

**Keywords:** Poverty alleviation, Conditional cash transfers, Women's leadership, Women's agency, Buenaventura, Households’ socioeconomic dynamics

## Abstract

This study provides detailed information on socially disadvantaged women and their households in Buenaventura, Colombia. A representative sample for 400 women aged between 18 and 70 was prepared and their living conditions were accessed based on the sociodemographic data and economic and social dynamics of their households. It gives special attention to access to poverty alleviation programs – conditional cash transfers - and forms of agency propelling women towards social change. The data were collected in July 2019 through in-person interviews. The database can greatly contribute toward further comparative research, informing public policy, and more broadly, widening the universe of statistical data on Colombia and other Latin American countries by providing quantitative data on welfare dependence, intragenerational pathways of social mobility, and changes in reproductive strategies and the political culture of women with respect to urban precarity. Fundación WWB Colombia and Universidad Icesi sponsored data collection as part of the project entitled “Analysis of the relationships between gender constructions and welfare programs in southwest Colombia”.

## Specifications Table

**Table:** 

Subject	Poverty reduction
Specific subject area	Cash transfer programs
Type of data	Text, Dummy, and Metrics Values
How data were acquired	In person surveys
Data format	Raw
Parameters for data collection	The unit of analysis are women from poor urban areas of Buenaventura, 18 to 70 years, beneficiaries or not of conditional cash transfers governmental programs in Colombia.
Description of data collection	The research team hired “Cifras & Conceptos” to carry out in person surveys to women in Buenaventura (Colombia). Data described is a representative sample from the seven poorest areas in the city.
Data source location	Cali -Colombia
Data accessibility	With the article http://oemcolombia.com/subsidios-y-construcciones-de-genero/

## Value of Data

• The data collected during the survey provide detailed information concerning living conditions within the households of poor women in Buenaventura, Colombia. The database goes beyond economic aspects, capturing useful information on social conditions related to the use of time, forms of reciprocity and care, social capital, and women participation in formal or informal networks (e.g., educational services, community-based processes, or forms of activism).
• The data provides a rigorous overview of financial changes across generations within one single household, thus mapping their financial inclusion and access to assistance programs aimed at poverty alleviation and enhancing the economic productivity of disadvantaged women.
• The data can be used in further comparative research to inform public policy and more broadly, widen the range of statistical data on Colombia. They can fill the gap in the previously incomplete or nonexistent information in scientific literature on cash conditional transfers programs, financial inclusion, and strategies of socially disadvantaged women with respect to urban precarity in Latin America.
• The database allows to critically assess the impacts of assistance programs on lives of socially disadvantaged urban women and their families. It provides quantitative data related to welfare dependence, intragenerational pathways of social mobility, and changes in the reproductive strategies, political culture, use of time, and life projects of the socially disadvantaged women.
• This study develops an original methodology for collecting and analyzing new empirical data within the global and interdisciplinary growing field of studies that explore the impacts of poverty reduction programs. Additionally, the design of the survey can serve as a springboard and a model for capturing quantitative data on social phenomena traditionally explored qualitatively in the existing literature.

## 1 Data Description

The data were collected in July 2019 through a single round of in-person interviews of 400 women aged between 18 and 70 living within seven localities in Buenaventura, Colombia. A structured questionnaire was designed by the research project directors and administered by trained pollsters. Two main topics were covered, namely, transgenerational dependency on cash monetary transfers and agency mechanisms.

The interviewed women answered questions based on their socioeconomic backgrounds, access to health services, transgenerational use of cash and monetary transfers, community and political participation, entrepreneurship, household decision-making processes, financial inclusion, leadership, autonomy, and agency. The questionnaire included 56 questions from which 862 variables were systematized. On average, the completion of a survey took approximately 40 min.

General socioeconomic characteristics of participants are presented in Table 1 with the following variables: ethnic identity, age group, household chiefdom, education attainment, income level, access to health services, and marital and occupational status. Moreover, relevant data allowing the estimation of women's personal autonomy in relation to household composition is provided. Thus, factors such as number of children, age of first pregnancy, dependency ratio, and household size are included.

**Table 1:** Socioeconomic characteristics of women.

	Percentage			
Demographics and income				
Household Head	58,5			
Black Ethnicity	89,5			
Less than 1 minimum wage of monthly Income	61,7			
*Level of education attainment*				
None	2,0			
Primary (complete or incomplete)	22,4			
Secondary (complete or incomplete)	52,8			
Tertiary (complete or incomplete)	22,5			
*Health services(1)*				
Subsidized system	50,0			
Non subsidized system	46,5			
None	1,7			
Marital status				
Single	54,9			
Couple	44,8			
Job conditions				
Homemaker	41,6			
Partial or full time worker	21,5			
Self-Employed	20,3			

	Mean	Std. Dev.	Min	Max
Other Characteristics				
Average woman age	44,12	16,95	18	89
Household composition				
Size (Average number of members)	4,47	1,80	1	11
Dependence ratio (2)	0,36	0,24	0	1
Age of first pregnancy	20,32	4,66	14	40
Mean number of children	2,70	2,31	0	22

Note: (1) In the subsidized health regime, beneficiaries do not pay for the service, while in the non-subsidized regime, people pay a monthly fee to access health services.

(2) Dependence ratio: (children under 12+adults over 65)/total members of the household.

Table 2 presents the details of the participants’ access to financial services and products and entrepreneurship opportunities and challenges faced by them in the process. It also shows the percentage distribution of the participants’ experiences on household decision-making processes, leadership, and public participation. Decisions refer to sexual and reproductive health, use of time, household financial decisions, and care responsibilities.

**Table 2:** Descriptive statistics on financial inclusion, entrepreneurship, leadership and agency.

	Percentage (%)
*Financial Inclusion*	
Households with savings	29,9
Households with some loan	29,5
Households with savings outside financial institutions	81,7
Households with loans by banks	70,9
	
*Entrepreneurship*	
Women that have carried out any business or entrepreneurship idea	38,0
Nowadays, Does the business continue?	72,0
	
*Main reasons for ending the business*	
Lack of money or resources	47,9
Security problems in the area	17,8
Did not have enough earnings or income	15,9
	
*Leadership and agency*	
	
*Decision-Making*	
Women who do not make decisions about the household	37,33
Women who do not make decisions about themselves	45,7
Women who do not make any decisions	30,4
	
*Main decisions made by women only*	
What the household buy for food	54,2
How the money is spent at home	45,5
Whether or not to attend meetings and parties	43,5
	
*Leadership and public participation*	
Women who know spaces for democratic participation	65,5
*Main spaces for democratic participation used*	
Religious organizations	51,8
Citizen Oversight (1)	29,7
Afro-Colombian organizations	25,6

Note: (1) Citizen oversight is a formal participation mechanism for reviewing government activities.

Table 3 shows number of surveys planned and effectively carried out in each locality or commune. The actual sample was, in most cases, very similar to the planned sample. In any case, the very small changes observed in the table did not affect the representativeness of the sample. Moreover, the sample size was as initially planned.

**Table 3:** Sample size.

Commune	Planned sample	Actual sample
3	40	33
4	46	49
5	54	56
7	55	56
9	51	48
10	62	67
12	91	91
Total	400	

The statistics department of Cifras & Conceptos (C&C), the firm we hired to collect the data, employed a map of each locality or commune to designate the specific areas where interviews had to be conducted. They established a goal of surveys for each block in each commune as shown in Table 4. If at the end of an initial block the sample per block target was not realized (i.e., maximum eight interviews per block), the pollsters would begin applying surveys in a subsequent block based on the numeration previously established on the map [1].

**Table 4:** Number of surveys per block and household.

Item	Number of surveys
Maximum surveys per side of block	2
Maximum surveys per block	8
Maximum surveys per dwelling	1
Maximum surveys per household	1

As a supplementary file, we provide the questionnaire in a Microsoft Excel format. It has eight sections and 56 questions. Finally, the database and codebook are available as Microsoft Excel files using the following link: http://oemcolombia.com/subsidios-y-construcciones-de-genero/.

## 2 Experimental design, materials, and methods

Information gathering was done by a hired firm, namely, C&C. It was necessary to avail services of an experienced ally like them owing to high level of insecurity caused by the criminal activities in Buenaventura [2]. This fact implied the need to properly organize the timings for the interviewers’ visits to the households to reach both working and nonworking women in the surveyed areas. C&C had key contacts in the areas, which allowed them to complete the interviews without any trouble.

Surveys were conducted to enquire 400 women aged from 18 to 70; habitual residents of seven communes were carefully selected considering those with the highest levels of poverty. The age range was 18–70 years for legal reasons (children under 18 years of age can only be interviewed after their parents’ permission) and we wanted to prevent over-participation of retired women.

With respect to the representativeness of the sample, the 400 women from 18 to 70 years of age interviewed approximately represent 98.522 women living in 59.526 households (composed by 233.853 people). According to the technical report we received from C&C, the sample size allows for the estimation of the total population with the error margin of 5.5% and a 95% level of confidence. The formula to arrive at these numbers is

$$ESrel=\frac{\sqrt{\left(1-\frac{n}{N}\right)\;\frac{P\left(1-P\right)}{n}\;Ed}}{P}$$

where,

ESrel is the standard relative error

*N* is the size of the universe (228.952 individuals)

*P* is the probability of occurrence

$Ed=\;\frac{Var\;(conglomerate)}{Var\;(MAS)}$ is the conglomerates effect

*n* is the sample size.

Considering an adequate balance between the sample size, results’ precision, and expected disaggregation levels, the C&C team defined a sample size of 400, which allows a maximum relative standard error of 5.5% for observed frequencies with the minimum population of 50% (probability of occurrence).

In-person interviews were conducted by nine pollsters in one week in July 2019, and they were supported by two supervisors. The supervision consisted of three steps: direct monitoring of 10% of the surveys of each pollster, critical review recording of 100% of the collected materials, and reverification of 10% of the surveys of each pollster.

## Ethics Statement

The survey was conducted with the informed consent of all the women who joined the study. A trained local team of pollsters from C&C contacted the households and informed all research participants about the aims of the research, politics of privacy, and protection of their data. All questions and doubts were clarified and answered. This was followed by obtaining explicit agreement and consent to participate in the research from all those involved in the process. All procedures of data collection and analysis followed the ethical guidelines formulated in the international regulations of ICC/Esomar Code of Ethics and the Colombian Law for data protection. mmc1.xml

## Declaration of Competing Interest

The authors declare that they have no known competing financial interests or personal relationships which have or could be perceived to have influenced the work reported in this study.

## Appendix Supplementary materials

Image, application 1
